# Temporary childbirth migration and perinatal healthcare in rural Maharashtra, India

**DOI:** 10.1016/j.jmh.2025.100322

**Published:** 2025-03-29

**Authors:** Rachel Murro, Alison M. El Ayadi, Rutuja Patil, Dhiraj Agarwal, Sanjay Juvekar, Juliana Kim, Nadia G. Diamond-Smith

**Affiliations:** a Department of Epidemiology and Biostatistics, University of California, San Francisco, 550 16th St 2nd Floor, San Francisco CA 94158 USA; b Department of Obstetrics, Gynecology, & Reproductive Sciences, University of California, San Francisco, 550 16th Street, #3745, San Francisco CA 94158 USA; c Vadu Rural Health Program, KEM Hospital Research Centre, Sardar Moodliar Road Rasta Peth, Pune 411 Maharashtra India; d University of California, Berkeley, 2000 Carleton Street #2284 Berkeley, CA 94720-2284, USA

**Keywords:** Maternal health, Childbirth, Temporary migration, Internal migration, Care continuum, Antenatal care, Postpartum care

## Abstract

**Introduction:**

Maternal health in rural India has improved due to national initiatives, but inadequate healthcare provision persists across most sociodemographic groups. The connection between perinatal care and the widespread practice of Temporary Childbirth Migration (TCM)—returning to one's natal home for delivery and the postpartum period—remains unexplored.

**Methods:**

Cross-sectional data on migration and health visits were collected from a sample of 1288 women in the Vadu Health and Demographic Surveillance Site (HDSS) (Western Maharashtra) who gave birth in 2018–2022. Childbirth migration (exposure) was analyzed in three ways: binary (any migration), continuous (duration of stay), and multidimensional (duration of stay and change of provider). Outcomes included number of health visits – both facility check-ups and home visits by community health workers – and early antenatal care initiation. Multivariate poisson, negative binomial and logistic regressions were used. Sensitivity analyses checked for recall bias, the influence of migration distance, and model misspecification.

**Results:**

Migrators and non-migrators had similar outcomes in early antenatal care initiation, facility visits (before and after pregnancy), and prenatal community health worker visits. Migrators had fewer postnatal community health worker visits (IRR = 0.80; 95 % CI 0.70–0.92). Among migrators, longer natal village stays were associated with fewer community health worker visits in the prenatal period (IRR = 0.92; 95 % CI 0.88–0.96) but not postnatally (IRR = 1.03; 95 % CI 1.00–1.07). Women who switched to a new provider upon arriving in their natal village had fewer facility-based prenatal (IRR = 0.86; 95 % CI 0.78–0.96) but more postnatal visits (IRR = 1.41; 95 % CI 1.06–1.87), regardless of how long they stayed.

**Conclusion:**

For women who return to their natal home for childbirth, duration of stay and changing providers upon arrival are linked to differences in receipt of maternal healthcare. Increased attention to the needs of mobile women during the perinatal period is necessary to ensure they can participate in key birth customs while receiving adequate healthcare.

## Highlights

• Women who return to their natal home for childbirth may miss out on critical facility and home visits throughout the peripartum period.
• Postpartum women who return to their marital home shortly after childbirth may face the greatest interruptions, particularly in home visits by community health workers.
• Care continuity during the postnatal period is critical; women who find a new provider near their natal home fare better than those who do not after delivery.

## Introduction

Across Asia, pregnant women often return to their natal homes during pregnancy or postpartum, a practice known as "temporary childbirth migration" (TCM). This tradition can elicit family support during a critical period but may disrupt perinatal care. Evidence on the practice and its consequences is limited yet could inform opportunities for optimizing maternal and neonatal health due to these potentially conflicting effects. In Japan, *Satogaeri Bunben*, where women return home in the third trimester and stay postpartum, is believed to enhance maternal mental health and bonding and reduce postpartum depression, though there's no quantitative evidence to support these claims (Okano, 1998; Tamaki et al., 1997; Yoshida et al., 2001). Conversely, the Chinese practice of *peiyue care*, starting in the second trimester, has been linked to fewer and later antenatal care visits (Ding et al., 2020; Zong et al., 2018). It is consequential to understand this phenomenon in Asian countries with poorer performance on maternal and neonatal health indicators, such as India. As of 2021, the Maternal Mortality Rate in India was 103 per 100,000 live births (Trends in maternal mortality 2000 to 2020: estimates by WHO, UNICEF, UNFPA, World Bank Group and UNDESA/Population Division 2023) and the neonatal mortality rate was 18 per 1000 live births (United Nations Children's Fund, World Health Organization, World Bank Group, United Nations Department of Economic and Social Affairs 2023). Coverage gaps and inequities are directly related to morbidity and mortality; thus access to care throughout pregnancy, delivery, and the postpartum period is vital to ensuring the health of women and their newborns.

Only one study has examined how TCM is linked to maternal and child health in India. Among women from two states, Madhya Pradesh and Bihar, migration was not a strong predictor of perinatal health visits overall, though women from Bihar who stayed in their natal home for longer tended to have more prenatal health visits (Diamond-Smith et al., 2024). Evidence on the impact of women's migration broadly (not for childbirth) on maternal and child health outcomes in India is also mixed. However, migrants, especially recent migrants, tend to be less likely to achieve key perinatal care indicators when compared to non-migrant women, including attaining the recommended ANC visits, delivering in a facility, and receiving timely postnatal care (Heaman et al., 2013; Jeyashree et al., 2018; Kusuma et al., 2013). Understanding how TCM may impact access to different types of maternal and newborn infant care is crucial for targeting services effectively.

Pregnancy-related care coverage in India varies significantly by type of care. According to the latest Demographic and Health Survey (NFHS-5), 59 % of women had four or more antenatal visits, with 70 % starting care in the first trimester. Postnatal check-ups within two days were received by 61 % of women and 82 % of newborns (International Institute for Population Sciences (IIPS) 2021). Disparities exist by socioeconomic status, demographics, state, and urban/rural areas. In rural areas, 55 % of women made the minimum antenatal visits, compared to 69 % in urban areas. Women in rural areas, with more children, lower income, and less education generally fare worse on most maternal and child health indicators. Maharashtra, the focal state for this study, meets or exceeds national averages but still shows these disparities (International Institute for Population Sciences (IIPS) 2021). Additionally, these indicators fail to fully capture the extent of comprehensive, high-quality care and likely overestimate care coverage. Recent data in Maharashtra indicate that high-quality antenatal, delivery, and postnatal care rates are 31 %, 85 %, and 29 %, respectively (Singh et al., 2022).

To address gaps in coverage, the government of India employs Community Health Workers (CHWs) through initiatives such as the National Rural Health Mission (NRHM), part of a comprehensive national maternal and child health strategy. Auxiliary Nurse Midwives (ANMs), Accredited Social Health Activists (ASHAs) and Anganwadi Workers (AWWs) all provide village- and home-based care for vulnerable populations. ASHAs are volunteers and receive performance-based incentives for care provision. They play a particularly crucial role in supporting pregnant and postpartum women and their newborns, offering pregnancy counseling, antenatal exams, nutritional assistance, and access to healthcare facilities (Ministry of Health and Family Welfare n.d; National Rural Health Mission 2011). ASHAs are linked to increased ANC visits and facility deliveries, with some evidence suggesting a dose-response relationship and reduction in neonatal mortality (Agarwal et al., 2019a; 2019b; Tripathy et al., 2016). ASHAs and other CHWs typically cover catchment areas comprising around 1000 people. Women who temporarily relocate to their natal homes for childbirth may be more familiar with the ASHA in their husband's home area, while the ASHA in their natal home may be unaware of their service needs or not have the resources to serve these additional women in their coverage area (Acevedo-Garcia et al., 2012).

Gaps in coverage for perinatal care – both facility-based and home visits – pose a threat to maternal and child health, particularly in low-income countries. Investigating the upstream predictors of these gaps is thus critical. This paper will examine how migration during the perinatal period is associated with number and timing of health visits. A greater understanding of how childbirth migration is connected to care continuity will be a first step towards paving the way for programs and policies tailored to the needs of women who are mobile during the pregnancy and postpartum period.

## Materials and methods

### Data

Data for this study was collected within the Vadu Health and Demographic Surveillance System (HDSS) in Pune district, Western Maharashtra, India. Established in 2002, the Vadu HDSS longitudinally monitors 22 villages with a total population of approximately 180,000 individuals. Initially biannual, data collection on pregnancies, births, marriages, migrations, and deaths transitioned to annual in 2018.

For this study, additional cross-sectional data collection was integrated into routine HDSS procedures to investigate temporary childbirth migration. Data collection took place between November 2022 and March 2023. The sampling frame (*N* = 2270) was the total number of Marathi-speaking women who gave birth in the Vadu HDSS site in 2018 or later (as reported during routine HDSS activities). We excluded births earlier than 2018 given potential recall bias if respondents reported on pregnancies from more than 4–5 years in the past.

As this study acted as a pilot study for a larger, longitudinal project and we lacked resources to survey all 2270 women in the sampling frame, we estimated that a sample size of ∼1250 women would provide reasonable power to allow detection of care differences. In post-hoc analyses, we assessed minimal detectable effect sizes for models examining the role of a binary migration exposure on the four different visit number outcomes. We calculated that we had 80 % power on a two-sided 0.05 level test to detect an absolute difference in visit numbers (delta) between those who migrated and those who did not between 0.25 and 0.60, depending on the model outcome (0.59 for pre-delivery ANC visits, 0.25 for pre-delivery ASHA visits, 0.43 for post-delivery ANC visits, and 0.32 for post-delivery ASHA visits). This suggests that the study may have been sufficiently powered to detect a small/medium difference for the ASHA visit outcomes but was potentially underpowered for detection of differences in ANC visit outcomes.

Probability proportionate to size (PPS) sampling (Lohr, 2010) was employed, with participants randomly selected within each village based on the village's number of annual births. This protocol ensured that the sample was geographically representative of the full HDSS site, given that the 22 villages differ in population size, socioeconomic status, and care access and quality characteristics. Trained Field Research Assistants (FRAs) obtained informed consent and administered structured questionnaires in Marathi using Android phones and the Survey Solutions platform (The World Bank, Washington DC). Data quality was ensured through continuous monitoring by an internal quality assurance team and researchers at UCSF. Fieldworkers participated in regular meetings, trainings, and recontacting efforts to address missing or erroneous data.

## Key measures

### Health visits

Participants were asked two open-ended questions to determine the number of health facility visits they made before and after delivery, focusing on their most recent pregnancy (referred to as the “index” pregnancy). They also reported the date of their first health facility visit during pregnancy. The approximate gestational age at the first visit was calculated using this date and the participant's reported approximate date of their last menstrual period preceding the index pregnancy. WHO (WHO 2016) and Indian government (Guidance Booklet on Maternal Health: Community Health Officers n.d) recommendations advise that women should receive their first antenatal care check-up in the first trimester. Participants also reported the total number of visits by ASHAs that they received both before and after delivery of the index pregnancy.

### Temporary childbirth migration

Participants were asked whether they had spent more than two weeks at their natal home during the index pregnancy or postnatal period. Those who responded yes provided departure and return dates. All participants provided their delivery and approximate last menstrual period (LMP) dates, which were used along with the departure and return dates to calculate days spent at their natal home during the pregnancy and the postnatal period. Participants reporting any migration also provided the name of their natal village. FRAs used Google Maps to estimate approximate migration distances between husband and natal home.

The definition of temporary childbirth migration (TCM) varied by analysis. For binary exposure models related to prenatal or postnatal care, TCM was indicated by any reported migration before or after delivery. Models examining migration duration defined TCM by months spent in the natal village before or after delivery. Participants who migrated were also asked if they changed healthcare providers upon arrival at their natal village.

### Sociodemographic and pregnancy-related characteristics

Participants reported their current age and pregnancy history, including lifetime number of births, miscarriages, and abortions. Marital status, occupation, household income and educational attainment were obtained via multiple choice questions. Additional linked data from previous routine HDSS surveillance included religion, the highest education level attained within the participant's household, and household type (nuclear or multigenerational). Participants also reported any health conditions during pregnancy and complications during delivery.

### Statistical analysis

Data management and analysis was performed in StataSE 17.0 (StataCorp, College Station, TX). Summary statistics of sociodemographic characteristics and care coverage were calculated for the full sample, and by binary TCM exposure. Univariate tests of differences by TCM exposure were conducted using Chi-square tests. The independent relationship between TCM and care coverage was examined via a series of multivariate regression models, described below.

### TCM as a one-dimensional exposure: any migration

Early initiation of antenatal care (within 12 weeks gestational age) was modeled using a multivariate logistic regression. Either multivariate poisson regression or negative binomial regression was used to model the number of ASHA home visits and facility check-ups before and after delivery, given the right skew of these discrete count outcomes. Poisson regression was utilized when equal dispersion of the mean and variance of the dependent variable was observed (Yang et al., 2007); negative binomial models were used in cases of moderate overdispersion. Models predicting prenatal and postnatal visits used any migration during the prenatal or postnatal period as the primary exposure respectively.

### TCM as a one-dimensional exposure: duration of stay

To assess the potential role of migration duration, multivariate regressions were conducted to model each visit outcome using length of stay in natal village (in months before or after delivery, according to the outcome). All models with primary exposure of duration of stay excluded people who did not migrate during the period of interest. Poisson or negative binomial regressions were used according to model fitness.

### TCM as a multi-dimensional exposure: the role of duration and provider change

We also investigated a third domain: provider change upon arrival to the natal village. We ran multivariate regressions for each facility visit indicator with two primary TCM exposures: duration of stay and provider change. We examined predictors of visit numbers overall and in each location (natal vs. marital home) to contextualize findings. Models also included an interaction term between duration and provider change to check for effect heterogeneity. Models were necessarily limited to migrators only; non-migrators were not asked if they changed providers so their inclusion in the multidimensional models would induce structural positivity violations (Petersen et al., 2012). Poisson or negative binomial regressions were used according to model fitness.

Care-seeking behaviors of migrators only, including visit numbers (home and facility-based) disaggregated by location (natal vs. marital home) were summarized to contextualize findings of all multivariate regressions.^1^

All models were adjusted for participant age, past births (categorical), education level (categorical), religion (Muslim, Hindu, Buddhist or Other), any pregnancy complications (binary), household income (sample quantiles), and household educational attainment (categorical). Covariates were determined a priori based on a conceptual framework of temporary childbirth migration and healthcare coverage. We conducted sensitivity analyses for all models (1) excluding migrators who traveled 5 km or less and (2) excluding women whose child was born more than two years before their interview. We also constructed alternatives to each visit number model using a multi-level categorical outcome variable to assess sensitivity to changing definitions of key outcomes. We considered 95 % confidence intervals that excluded the null test value evidence of statistical significance.

### ETHICS approval

Ethical approval was obtained from the KEMHRC Institutional Ethics committee (letter KEMHRC/RVM/EC-1899 dated 29th September 2022) and the University of California, San Francisco (22–36,484). All participants gave informed consent to participate in the study.

## Results

### Descriptive and bivariate findings

1288 women met the eligibility criteria, consented to participate, and were included in the final sample. Of these individuals, most were between ages 19–24 (34.0 %) or 25–29 (44.5 %), currently married (99.8 %), Hindu (92.5 %), and did not work outside the home (93.6 %). Nearly half of participants (47.0 %) were nulliparous before their index birth, while 43.5 % had given birth once prior. Most had completed secondary (23.5 %) or higher secondary (35.4 %) school or had graduated (26.4 %). All participants lived in a multigenerational household (Table 1).

**Table 1:** Characteristics of Vadu HDSS participants with a recent birth, 2022–2023 (*N* = 1228).

	Total (*N* = 1228)	Any temporary childbirth migration† (*N* = 995)	No temporary childbirth migration (*N* = 233)	p-value††
	Frequency (%)			
Individual characteristics				
Age, years				.013*
19–24	418 (34.0)	341 (34.3)	77 (33.1)	
25–29	547 (44.5)	453 (45.5)	94 (40.3)	
30–34	316 (17.6)	171 (17.2)	45 (19.3)	
35+	47 (3.8)	30 (3.0)	17 (7.3)	
**Marital status**				.401
Currently married	1225 (99.8)	992 (99.7)	223 (100.0)	
Unmarried	3 (0.2)	3 (0.3)		
**Parity**				<0.001⁎⁎
Index was first birth	577 (47.0)	490 (49.3)	87 (37.3)	
2 births	534 (43.5)	427 (42.9)	107 (45.9)	
3 or more births	117 (9.5)	78 (7.8)	39 (16.7)	
**Religion**				.195
Hindu	1066 (92.5)	872 (93.3)	194 (89.9)	
Muslim	27 (2.3)	20 (2.1)	7 (3.2)	
Buddhist	49 (4.3)	35 (3.7)	14 (6.4)	
Other	11 (1.0)	8 (0.9)	3 (1.4)	
**Education**				<0.001⁎⁎
Primary or less	122 (9.9)	69 (6.9)	53 (22.8)	
Secondary	289 (23.5)	220 (22.1)	69 (29.6)	
Higher secondary	435 (35.4)	367 (36.9)	68 (29.2)	
Graduate	324 (26.4)	287 (28.8)	37 (15.9)	
Post-graduate	58 (4.7)	52 (5.2)	6 (2.6)	
**Occupation**				.375
Housewife	1149 (93.6)	928 (93.3)	221 (94.9)	
Works outside the house	79 (6.4)	67 (6.7)	12 (5.2)	
**Household characteristics**				
**Monthly income (INR), sample quantiles**				.001⁎⁎
Lowest	268 (21.8)	195 (19.6)	73 (31.3)	
Lower	389 (31.7)	330 (33.2)	59 (23.3)	
Middle	188 (15.3)	156 (15.7)	32 (13.7)	
Higher	159 (13.0)	136 (13.7)	23 (9.9)	
Highest	224 (18.2)	178 (17.9)	46 (19.7)	
**Highest household education level**				<0.001⁎⁎
Primary or less	69 (5.6)	41 (4.1)	28 (12.0)	
Secondary	136 (11.1)	100 (10.1)	36 (15.5)	
Higher secondary	419 (34.1)	341 (34.3)	78 (33.5)	
Graduate	445 (36.2)	381 (38.3)	64 (27.5)	
Post-graduate	159 (13.0)	132 (13.3)	27 (11.6)	
**Household type**				n/a
Joint (multigenerational)	1228 (100.0)	995 (100.0)	233 (100.0)	
**Health coverage**				
First prenatal facility visit <12 weeks GA	1156 (97.6)	946 (97.7)	220 (96.9)	.49
8+ prenatal facility visits	985 (80.2)	814 (81.8)	171 (73.4)	.004⁎⁎
4+ postnatal facility visits	175 (14.3)	134 (13.5)	41 (17.6)	.105
Any prenatal ASHA home visits	1013 (82.5)	829 (83.3)	184 (79.0)	.116
Any postnatal ASHA home visits	886 (72.2)	720 (72.4)	166 (71.2)	.732
Facility delivery	1219 (99.3)	992 (99.7)	227 (97.4)	<0.001⁎⁎
Changed providers upon arrival in natal village	n/a	203 (20.4)	n/a	

† Defined as a stay in natal village ≥2 weeks.

†† Obtained via Chi-square tests.

⁎ Significant at alpha <0.05.

⁎⁎ Significant at alpha <0.01.

81.0 % of the sample migrated to their natal home for at least two weeks, either while pregnant or shortly following delivery. Most migrators left while they were in their 7th month of pregnancy (20.6 %), 8th or 9th month of pregnancy (41.1 %) or within the week after delivery (23.0 %). Relatively few migrated earlier than the 7th month (8.5 %) or more than a week after delivery (7.2 %). Almost all returned to their husband's home when the baby was 1–2 months old (23.4 %) or 2–6 months old (68.8 %). Those who migrated tended to be younger, nulliparous prior to the index birth, more educated and wealthier. In-depth analyses of temporary childbirth incidence and its predictors in this sample have been documented in a separate publication (Diamond-Smith et al., 2025).

Table 1 also includes summary statistics for health indicators, including pre-delivery health facility visits (80.2 % ≥ 8 visits), post-delivery health facility visits (14.3 % ≥ 4 visits), early ANC initiation (97.6 % within 12 weeks), and ASHA exposure (82.5 % before delivery, 72.2 % after delivery). Nearly all participants delivered in a health facility (99.3 %). In bivariate analyses, those who migrated had higher coverage rates for pre-delivery health facility visits and facility delivery (81.8 % and 99.7 %) compared to those who stayed home (73.4 % and 96.9 %). There were no other marginal differences in care coverage by binary migration status. 20.4 % of all migrators reported changing to a new provider once they arrived in their natal home.

### Multivariate analyses: any migration as exposure

Table 2 shows results from models of health visit counts/timing (dependent variables) as a function of *any* childbirth migration (independent variable). After adjusting for participant age, parity, education, religion, and pregnancy complications and household income and educational attainment, we did not find significant evidence of a non-spurious association between any migration and health facility visits before delivery (IRR = 1.01; 95 % CI 0.97, 1.05), nor with odds of initiating ANC care by 12 weeks’ gestation (OR = 1.27; 95 % CI 0.59, 2.74), nor health facility visits after delivery (IRR = 1.01, 95 % CI 0.88, 1.16). Migrating was also not associated with ASHA home visits during the prenatal period (IRR=0.95; 95 % CI 0.88, 1.02). However, migrating was associated with a reduced rate of postnatal ASHA visits (IRR = 0.80; 95 % CI 0.70, 0.92).

**Table 2:** Association between Temporary Childbirth Migration and Healthcare Coverage Indicators.

		aOR or aIRR (95 % CI)				
		First prenatal visit < 12 weeks GA	Prenatal facility visits	Postnatal facility visits	Prenatal ASHA visits	Postnatal ASHA visits
Binary migration exposure (*N* = 1228)	No migration (ref)	1.00	1.00	1.00	1.00	1.00
	Any migration ≥2 weeks	1.27 (0.59–2.74)	1.01 (0.97–1.05)	1.01 (0.88–1.16)	.95 (0.88–1.02)	.80⁎⁎ (0.70–0.92)
Continuous migration exposure†	Days migrated during prenatal period (*N* = 694)	.72 (0.57–1.10)	.99 (0.97–1.02)			1.03⁎ (1.00-1.07
	Days migrated during postnatal period (*N* = 990)			1.03 (1.00-1.07)	.92⁎⁎ (0.88–0.96)	

⁎ Significant at alpha <0.05.

⁎⁎ Significant at alpha <0.01.

† Sample for continuous models with continuous migration exposure only include only migrators (those who stayed in natal village ≥2 weeks during the prenatal or postnatal period, depending on the model outcome. aOR: adjusted odds ratio. aIRR: adjusted incidence rate ratio. Models adjusted for participant age, parity, religion, education, and complications during pregnancy, and for household income and highest household education level.

### Multivariate analyses: duration of migration exposure

Table 2 also shows results from models of health visit counts/timing (dependent variables) as a function of *duration of* childbirth migration (independent variable). Among migrators only, there was no independent association between duration of stay in natal village and facility visit indicators. However, those who stayed longer had lower rates of ASHA visits before delivery (IRR = 0.92; 95 % CI 0.88–0.96) and slightly higher rates after delivery (IRR = 1.03; 95 % CI 1.00–1.07)

Table 2 results were similar (direction of effect size and significance levels) to those obtained in sensitivity analyses that (1) excluded migrators who traveled 5 km or less and (2) excluded women whose child was born more than two years before their interview. All findings for count indicators (visit numbers) were robust to categorization of the outcome variable and remained substantively unchanged whether robust standard errors with poisson regression or negative binomial regression was used.

### Multivariate analyses: migration as a multidimensional exposure

**Fig. 1:**
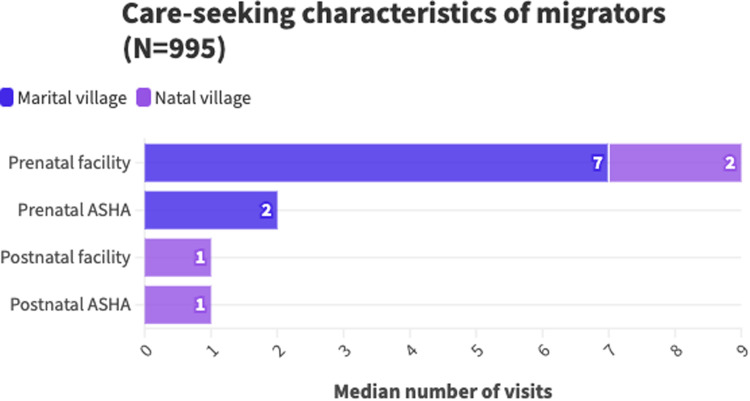
Care seeking characteristics of migrators(*N* = 995).

Fig. 1 presents summary statistics of care-seeking behaviors of migrators in the sample. Almost all migrators had at least one prenatal facility check-up while staying in their marital village (98.4 %, median = 7, IQR = 5–9) and in their natal village (90.1 %, median = 2, IQR = 1–3). 75.7 % of migrators had a postnatal facility check-up in their natal village (median = 1, IQR = 1–2) while only 19.1 % did in their marital village (median = 0, IQR = 0–0). Most migrators received an ASHA visit in their marital village before delivery (71.2 %, median = 2, IQR= 0–3); only 31.3 % received one in their natal village (median = 0, IQR = 0–1). 16.8 % received a postnatal ASHA visit in their marital home (median = 0, IQR = 0–0) and 62.4 % received one while staying in their natal home (median = 1, IQR = 0–2).^2^

Table 3 contains findings from models of facility visit counts (dependent variables) as a function of both duration of migration stay and provider change upon migrating (independent variables). Increasing time spent in natal home was associated with decreased prenatal facility visits in the marital village (IRR = 0.88; 95 % CI 0.85–0.91) but more in the natal village (IRR= 1.22; 95 % CI 1.17–1.27). There was no overall association between migration duration and total number of prenatal facility visits (IRR = 1.00; 95 % CI 0.97–1.02). Changing providers was inversely associated with prenatal facility visits in the marital village (IRR=0.82; 95 % CI 0.72–0.93) but not significantly related to those in the natal village (IRR=1.02; 95 % CI 0.84–1.23). Overall, there was a negative association between changing providers and total prenatal facility visits (IRR=0.86; 95 % CI 0.78–0.96). There was no association between duration of stay in natal village and postnatal facility visits, in either location, or overall (overall IRR=1.02; 95 % CI 0.98–1.06). Changing providers was positively associated with postnatal facility visits in the natal village (IRR=1.59; 95 % CI 1.20–2.10) and overall (IRR=1.41; 95 % CI 1.06–1.87), but not in the marital village (IRR=0.56; 95 % CI 0.19–1.66). There was no evidence of statistical interaction between duration of stay and provider change in any models.

**Table 3:** Association between length of stay in natal village, provider change, and facility visits, among migrators only (*N* = 995).

	aIRR (95 % CI)					
	Prenatal facility visits			Postnatal facility visits		
	Total	In marital village	In natal village	Total	In marital village	In natal village
length of stay (months)	1.00 (0.97–1.02)	.88⁎⁎ (0.85–0.91)	1.22⁎⁎ (1.17–1.27)	1.02 (0.98–1.06)	.99 (0.88–1.11)	1.02 (0.97–1.07)
provider change	.86⁎⁎ (0.78–0.96)	.82⁎⁎ (0.72–0.93)	1.02 (0.84–1.23)	1.41* (1.06–1.87)	.56 (0.19–1.66)	1.59⁎⁎ (1.20–2.10)
length of stayXprovider change (interaction term)	1.01 (0.97–1.06)	1.02 (0.95–1.09)	1.00 (0.93–1.08)	1.02 (0.94–1.11)	1.15 (0.82–1.61)	1.03 (0.94–1.11)

⁎ Significant at alpha <0.05.

⁎⁎ Significant at alpha <0.01 ^†^Sample for continuous models with continuous migration exposure only include only migrators (those who stayed in natal village ≥2 weeks during the prenatal or postnatal period, depending on the model outcome. aOR: adjusted odds ratio. aIRR: adjusted incidence rate ratio. Models adjusted for participant age, parity, religion, education, and complications during pregnancy, and for household income and highest household education level.

## Discussion

Understanding whether temporary childbirth migration plays a role in healthcare coverage is crucial for optimizing care delivery, particularly in rural India, where perinatal health checkups lag behind global standards (International Institute for Population Sciences (IIPS) 2021). Considering the emphasis on home visits to improve maternal and child health outcomes, it is also imperative to investigate challenges in service delivery within the home visit network. Our analysis found that TCM is very common, and migrators generally have comparable care coverage to non-migrators, aside from postpartum home ASHA visits, which are significantly lower among migrators. We identified groups of migrators at greatest risk for poor care coverage. Prenatal ASHA visits were particularly low among women who returned to their natal home earlier in pregnancy (longer duration of stay before childbirth). Duration of stay was generally not associated with number of facility visits, but changing providers was linked with care coverage. Those who changed providers upon arriving to their natal village fared worse when it came to prenatal facility visits, but better for postnatal facility visits.

>80 % of women in this study migrated to their parents’ home during pregnancy or post-delivery for at least two weeks. This is significantly higher than the 38 % prevalence found in preliminary research in India (Diamond-Smith et al., 2024), but close to the 50–70 % found in urban Japan (Takahashi and Tamakoshi, 2014; Tamaki et al., 1997; Yoshida et al., 2001). TCM is more common among higher-income, more educated women, as well as younger women and those experiencing their first childbirth.

Given the dearth of literature (1) characterizing TCM and (2) describing its potential role in health outcomes, we lacked a blueprint to define an exposure that would be comprehensive, consistent, and programmatically relevant. Therefore, we developed three definitions with increasing specificity: migration as a binary indicator (any or none), migration defined by duration of stay in natal home, and migration defined by both duration of stay and provider change.

Migration as a binary domain showed no independent association with facility health visits during the prenatal or postnatal period. This is in contrast to findings among rural to urban migrants returning home in China (Zong et al., 2018), but aligns with findings from rural Madhya Pradesh and Bihar (Diamond-Smith et al., 2024), suggesting that participating in TCM may not affect number of perinatal contacts with healthcare providers. Similarly, we found no overall association between duration of stay and facility visits among migrators. Women leaving their husband's home earlier in pregnancy compensated by making more prenatal facility visits while residing in their natal home. There was also no association between longer postpartum stays and visit numbers. These findings suggest that on average, women typically continue to receive the same quantity of facility health checks across pregnancy and the postpartum period, regardless of when they return and how long they stay. These results can broadly be framed within the lens of “positive” vs. “negative” residential mobility (Cunningham et al., 2010); women who migrate to their natal home are driven by positive forces (better support, care, rest) and can thus be classified as “positive” movers. Prior research has shown that residential mobility during pregnancy and infancy are linked to poor health only among negative movers, who typically fare worse overall due to poorer socioeconomic and life circumstances (Tunstall et al., 2010).

However, facility visit coverage was heterogenous according to whether migrating women reported finding a new provider upon return to their natal home. Migrating women who changed providers had significantly fewer prenatal health check-ups, regardless of their duration of stay. This was exclusively driven by decreasing check-ups in the marital village, which was *not* balanced out by an increase in the natal village. These findings were surprising; fewer than 3 % of women in this sample reported seeking better medical care as a reason for migrating (Diamond-Smith et al., 2025). Almost all migrated due to cultural tradition or belief they would receive better rest and care in their natal home. It is thus unlikely that poor perceptions of medical care quality near their husband's home drove women to migrate (reverse causation). Rather, we theorize that women planning to switch providers upon returning to their natal home may view the new provider as their primary one, foregoing prenatal facility visits before migration. This perception could be due to several factors. The provider seen during the natal home stay often delivers the baby, and women may feel more comfortable with them due to prior interactions or positive experiences of other natal family members. Research in Northern India indicates that women with close ties to their natal family rely heavily on their mother for care and guidance when traveling outside the home (Bloom et al., 2001), suggesting that women may prefer to make prenatal health facility visits when accompanied by their mother, thus reducing the facility visits while in their husband's home.

In contrast to findings about pregnancy visits, migrating women who changed providers had more overall postnatal health check-ups compared to those who didn't change provider. This was driven exclusively by an increase in check-ups while residing in the natal village. The potential protective effect for postnatal care is straightforward; women who do not find a new provider near their natal village, when unable to visit their prior provider who was located near their marital village, are unlikely to receive postnatal care there. These findings reflect existing evidence on internal migrants broadly, suggesting that recent mobility is linked to poorer healthcare utilization due to difficulty continuing care at a new facility (Kusuma et al., 2013). It's possible that the social support offered by a particularly involved natal family – one that supports the woman in scheduling and attending postnatal visits – is particularly important during the postnatal period. Given that over 55 % of women in this sample stayed in their natal village for more than two months after delivery, and 21 % for one to two months (Diamond-Smith et al., 2025), this would apply to nearly all migrants in this setting.

We found no evidence that migration as a binary domain was significantly associated with a change in prenatal ASHA visits; however, among those who did migrate, a longer natal home stay before delivery was associated with a decrease in ASHA visits. Few migrators saw an ASHA before delivery in their natal village (31.3 %), thus women who migrate earlier in pregnancy may be missing out on ASHA visits in their marital home. However, as most women who were in their natal home during pregnancy left close to their delivery date, we did not see an overall effect of migrating on prenatal ASHA visits.

Migrating was associated with an overall reduction in postnatal ASHA visits. This is contextualized by the site-specific statistics in Fig. 1; while 62.4 % of migrators saw an ASHA after delivery in their natal village, the median number of contacts was only 1, and only 16.8 % saw an ASHA upon going back to their marital home after delivery. Because women tended to stay in their natal home for several months after delivery and yet only received a median of one visit there, postnatal ASHA coverage was lower for migrating women. Still, among those who migrated, staying longer during the postnatal period was associated with marginally greater postnatal ASHA visits. This may suggest that women who remain in their natal village for longer are able to get on ASHA visit registries and receive care, while those who return shortly after birth have an interruption in home visits during a critical period. This aligns with qualitative findings in other states indicating challenges for community health workers in supporting pregnant women who temporarily relocate (Diamond-Smith et al., 2024). While the government of India launched a national, electronic Mother and Child Tracking System (MCTS) in 2009, the process of transitioning from hard copy registers is still ongoing in many states, including Maharashtra (Gera et al., 2015). Additionally, while e-registers are an important first step to ensuring continuity of care throughout the perinatal period, specific initiatives to link registers in different villages will be necessary to avoid gaps in care for women who chose to return to their natal village.

Timing of antenatal care initiation was independent of migration status and duration. This aligns with our expectations as fewer than 2 % of migrators returned to their natal village before the second trimester. This is also consistent with findings from literature across Asian where childbirth-related return typically commences in the second or third trimesters (Corbett and Callister, 2012; Yoshida et al., 2001; Zong et al., 2018), well after the recommended start of antenatal care.

Further research must investigate the mechanisms through which migration might be improving or interrupting care, and the extent to which changes impact care *quality.* Past studies have demonstrated that strong natal kin relationships are linked to greater autonomy, which in turn increases maternal healthcare utilization (Bloom et al., 2001). Given the high prevalence of migration after delivery observed here, future studies should explore the role of social support from the natal family in improving coverage, particularly during the postnatal period. Specific attention must also be paid to issues of delayed registration within ASHA networks and actions which can offer protection against care interruptions in the Indian context. For example, taking paper copies of medical records when migrating, as was observed in a sample of women returning home for childbirth in Thailand (Riewpaiboon et al., 2005), may limit care discontinuity.

### Limitations

This study has several limitations. Although we adjusted for reported health conditions during pregnancy, minor conditions may have been underreported, introducing possible bias through confounding by indication. We recognize the need for more robust analysis to properly adjust for pregnancy conditions, as well as to examine nuanced interactions between factors like duration, provider change, and distance traveled. Despite this, we present three models to attempt to identify subgroups at risk for care interruptions. Findings were robust to sensitivity analyses excluding migrators traveling <5 km, suggesting these “very close” cases did not significantly impacting our results.

Additionally, as this study used a retrospective cross-sectional design, recall bias may have influenced results. We did not examine multivariate associations between migration duration and ASHA visits disaggregated by location (marital vs. natal home), given concerns about the quality of granular data on reported ASHA visits. Findings presented here were robust to sensitivity analyses limited to recent births, but recall issues remain a concern as this topic requires self-report of detailed pregnancy experiences.

Lastly, this study was limited in scope. We lacked comprehensive quality-of-care indicators, which should encompass both evidence-based services and a positive care experience (as per WHO guidelines) (WHO 2016, 2017, 2022), and did not examine health outcomes (maternal and infant morbidity). To understand the *impacts* of temporary childbirth migration on care continuity, quality, and subsequent effects on health outcomes, longitudinal, prospective research is needed.

## Conclusion

This study shows that temporary childbirth migration is common and may be linked to interruptions in perinatal care visits – both facility-based and home visits. Whether this interruption leads to adverse maternal or newborn health outcomes remains uncertain, but further assessment of healthcare provision gaps is warranted. Rural health initiatives should consider creating formal systems to add temporary visitors to health registers based on information from local clinics, hospitals, and village leaders. Community health workers should receive training and support to visit women early in their pregnancy (first trimester), before they have migrated, and counsel women on the importance of creating a comprehensive, continuous care plan with providers and health checkups scheduled during their time at home and in their natal village.

This work was supported by the National Institutes of Health (R01HD107197).

## CRediT authorship contribution statement

**Rachel Murro:** Writing – original draft, Visualization, Methodology, Formal analysis. **Alison M. El Ayadi:** Writing – review & editing, Supervision, Project administration, Methodology, Investigation, Funding acquisition, Conceptualization. **Rutuja Patil:** Writing – review & editing, Project administration, Investigation, Funding acquisition, Data curation, Conceptualization. **Dhiraj Agarwal:** Writing – review & editing, Project administration, Investigation, Data curation. **Sanjay Juvekar:** Writing – review & editing, Investigation, Funding acquisition, Conceptualization. **Juliana Kim:** Writing – original draft, Data curation. **Nadia G. Diamond-Smith:** Writing – review & editing, Supervision, Project administration, Supervision, Project administration, Methodology, Investigation, Funding acquisition, Conceptualization.

## Declaration of competing interest

The authors declare that they have no known competing financial interests or personal relationships that could have appeared to influence the work reported in this paper.

## Footnotes

1 Multivariate logistic models were also estimated to examine predictors of changing providers among migrators (results are reported briefly but not shown in tables)

2 Among migrators, duration of stay and distance to natal village were statistically significant predictors of changing providers, though effect sizes for both were minimal (OR=1.00; 95 % CI 1.00-1.00 and OR=1.00; 95 % CI 1.00-1.01 respectively). No sociodemographic characteristics significantly predicted provider change (results of supplementary analyses not shown).
